# Structure alignment-based classification of RNA-binding pockets reveals regional RNA recognition motifs on protein surfaces

**DOI:** 10.1186/s12859-016-1410-1

**Published:** 2017-01-11

**Authors:** Zhi-Ping Liu, Shutang Liu, Ruitang Chen, Xiaopeng Huang, Ling-Yun Wu

**Affiliations:** 1 Department of Biomedical Engineering, School of Control Science and Engineering, Shandong University, Jinan, Shandong 250061 China; 2 Department of Computer Science, Stanford University, Stanford, CA 94305 USA; 3 Institute of Applied Mathematics, Academy of Mathematics and Systems Science, Chinese Academy of Sciences, Beijing, 100190 China; 4 National Center for Mathematics and Interdisciplinary Sciences, Chinese Academy of Sciences, Beijing, 100190 China; 5 University of Chinese Academy of Sciences, Beijing, 100049 China

**Keywords:** RNA-binding pocket, Local structure classification, Structural alignment, Network clustering, Structure motif

## Abstract

**Background:**

Many critical biological processes are strongly related to protein-RNA interactions. Revealing the protein structure motifs for RNA-binding will provide valuable information for deciphering protein-RNA recognition mechanisms and benefit complementary structural design in bioengineering. RNA-binding events often take place at pockets on protein surfaces. The structural classification of local binding pockets determines the major patterns of RNA recognition.

**Results:**

In this work, we provide a novel framework for systematically identifying the structure motifs of protein-RNA binding sites in the form of pockets on regional protein surfaces via a structure alignment-based method. We first construct a similarity network of RNA-binding pockets based on a non-sequential-order structure alignment method for local structure alignment. By using network community decomposition, the RNA-binding pockets on protein surfaces are clustered into groups with structural similarity. With a multiple structure alignment strategy, the consensus RNA-binding pockets in each group are identified. The crucial recognition patterns, as well as the protein-RNA binding motifs, are then identified and analyzed.

**Conclusions:**

Large-scale RNA-binding pockets on protein surfaces are grouped by measuring their structural similarities. This similarity network-based framework provides a convenient method for modeling the structural relationships of functional pockets. The local structural patterns identified serve as structure motifs for the recognition with RNA on protein surfaces.

**Electronic supplementary material:**

The online version of this article (doi:10.1186/s12859-016-1410-1) contains supplementary material, which is available to authorized users.

## Background

Protein-RNA recognitions play many fundamental and vital roles in crucial molecular interactions involved in biochemical reactions and signaling transduction [1, 2]. Revealing the RNA-binding structure motifs will provide insightful clues for deciphering the mechanisms of protein-RNA interactions and provide valuable knowledge for protein engineering, such as drug target design for silencing specific RNAs after transcription [3, 4]. Moreover, numerous diseases have been found to be related to dysfunctions of protein-RNA interactions due to RNA-mediated post-transcriptional gene regulation [5–7]. In this regard, deciphering the protein-RNA recognition code is central to physiology and disease [8, 9]. Recently, high-throughput approaches have made tremendous progress in the detection of protein-RNA interactions through chemical crosslinking and immunoprecipitation (CLIP) [10, 11]. The rich availability of protein-RNA interaction data provides the materials and opportunities to reveal their binding mechanisms. Some sequence patterns have been identified for protein-RNA recognition, such as RNA recognition motifs and zinc fingers [12]. The identification of binding motifs will provide a deep understanding of RNA-binding mechanisms, such as molecular characteristics and affinity distributions [9, 13]. These findings are often based on sequence-based approaches in CLIP data [14, 15] and focus on the nucleotide residue patterns from the side of RNAs [16]. To date, there are numerous unique RNA-binding domains listed in Pfam [17] and other databases of RNA-binding specificities such as RBPDB [18] and CLIPZ [19]. Some critical partners in the RNA interference of miRNA-binding functions, e.g., Dicer and Argonaute [20], also exhibit structural specificity and functional importance in protein-RNA interactions [21, 22]. Dissecting RNA recognition motifs from protein three-dimensional (3D) structures is still of great interest and importance.

Protein surfaces are among the major locations where RNA-binding events take place [23]. Pockets are one of the local structure patterns on protein surfaces and have proven to be concrete locations and detail-rich environments for many critical biological reactions, such as ligand binding [24, 25]. A protein-RNA binding pocket facilitates the local geometry for RNA packing and constructing protein complexes to perform certain functions [26]. Recent bioinformatics studies, such as PRNA [27], RNABindR [28], BindN [29], and PRINTR [30], have made substantial efforts to predict protein-RNA binding residues in proteins, but very few methods are available to identify the structure motifs that underlie the RNA-binding sites [2, 26, 31, 32], especially from the perspective of local protein surface regions. A pocket on a protein surface is among the typical structure patterns that provide the specific microenvironment needed to bind and regulate RNAs [33, 34]. The knowledge of RNA-binding structure motifs in the form of pockets will reveal the local structure groups and the underlying mechanisms involved in the recognition of RNA on protein surfaces. Identifying the structural patterns and physicochemical specificities of these binding pockets will greatly benefit downstream feature studies of protein-RNA interaction.

In this work, we conduct a large-scale analysis of the RNA-binding pockets on protein surfaces to identify the structural motifs and patterns of protein-RNA recognition. We first identify the RNA-binding residues in 3D protein structures and extract the surface pockets involved in the binding events from a compiled non-redundant protein-RNA complex dataset. Then, the local structure similarities among these RNA-binding pockets and the global structure similarities among their containing or parent proteins are measured via structure alignment methods. Using a similarity-network-based framework, the RNA-binding pockets and proteins are clustered into certain groups. The correspondences of RNA-binding pockets, domains and proteins are then investigated. The patterns of binding structure motifs and their functional implications in protein-RNA recognition are revealed accordingly.

## Methods

### Datasets

We download the RNA-binding protein complexes from the PDB [35]. There are 896 protein-RNA complexes available as of the beginning of our project. We eliminate complexes with protein and RNA sequences that are too long (i.e., more than 200 residues) or too short (i.e., 15 for protein and 5 for RNA). The homologous proteins are removed via BLASTclust [36] with sequence similarity of 25% in proteins and 60% in RNAs. In total, 158 non-redundant protein-RNA complexes remain for further analyses. All data and software used in this paper are available at our website: http://doc.aporc.org/wiki/PRNAclass.

The residues that exhibit binding between protein amino acids and RNA nucleotides are defined by Entangle [37]. Several interaction types, such as hydrogen bonding, electrostatic, hydrophobic and van der Waals interactions, are considered simultaneously in the binding residue identification. In this work, pockets on protein surfaces refer to empty concavities that the solvent can access. These concavities have mouth openings that connect their interiors with the outside bulk solution. We extract the surface accessible pockets using CASTp [38]. As many as 7664 pockets and their coordination are identified in these protein complexes. The RNA-binding pockets are defined as those pockets containing at least one RNA-binding residue detected by Entangle. These local structure pockets contain approximately 10 amino acid residues. After removing the very tiny pockets containing fewer than 4 residues, we obtain 786 RNA-binding pockets. We focus on the analysis of these pockets and their corresponding parent RNA-binding proteins (RBPs).

### Framework of classification

**Fig. 1:**
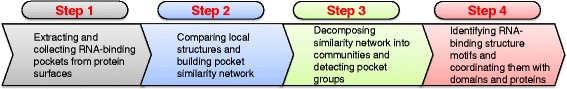
The framework for identifying structural RNA-binding motifs via pocket similarity network. **1** The RNA-binding pockets in proteins are extracted and collected from RBPs. **2** The pocket similarity network is constructed based on the similarities among these pockets calculated by our structure alignment algorithm SAMO. **3** The RNA-binding pockets are clustered into small groups with similar structures by decomposing the pocket similarity network into subnetwork modules via a community detection method. **4** The RNA-binding structure motifs and sequence patterns underlying the similar pocket groups are identified and analyzed

Figure 1 shows our proposed similarity-network-based framework for classifying the RNA-binding pockets into structural groups. A similar strategy is implemented to classify the parent RBPs after building a protein similarity network. First, all binding pockets are extracted from local protein surfaces. As described before, when a pocket contains at least one RNA-binding residue, we define it as an RNA-binding pocket. Second, we improve our structure alignment algorithm, SAMO (Structural Alignment by Multi-objective Optimization) [39], to precisely measure the similarities among these local structures and construct a pocket similarity network by connecting nodes that represent pockets. When there exists a significant similarity between two pockets, an edge is added to link them. The pocket similarity network is found to naturally conform to certain community structures. Third, we decompose the pocket similarity network into small clusters of RNA-binding pockets according to the network topologies. Last but not least, we identify the consensus binding pockets via multiple structure alignments as the structure motifs and local patterns of pockets in these pocket groups, with the hierarchical relationships across their parent domains and proteins.

### Comparing local structure similarities

To detect the structural similarities among RNA-binding pockets, we improve our non-sequential-order structure alignment algorithm SAMO to compare the local structures in an all-against-all manner.

SAMO formulates the structure alignment problem as a multiple-objective mixed integer programming problem. The first objective function is to minimize the total square distances of the aligned residues, and the second maximizes the total number of aligned residues. The optimization problem is solved by an iterative algorithm. The details of the SAMO algorithm for protein structure alignment can be found in [39].

For pocket similarity in the work, the structure alignment determined the similarity metrics of RMSD (root mean square deviation) between two RNA-binding pockets and the number of aligned residues,. Unlike [39], in this work, the measurement is then transformed into a *Q*-score as follows:

$$ Q=\frac{{\left({N}_{align}\right)}^2}{\left(1+{\left(\frac{RMSD}{R_0}\right)}^2\right){N}_1{N}_2}, $$

where *R*_0_ is a normalizing factor (set as 3.0), and *N*_1_ and *N*_2_ are the sequence lengths of the two pockets.

When applied to small local structures such as pockets, the structure alignment method tends to produce many false positives. To evaluate the statistical significance of the similarity in a population of alignments between any pair of local structures, we consider the similarity *P*-value instead of the RMSD of an alignment in SAMO [40–42]. Specifically, we obtain the pocket similarity significance through an empirical testing procedure. First, we randomly generate 200,000 pairs of pockets from the PDBSelect25 proteins [43]. These proteins have no obvious sequence similarities with each other [44]. Then, the alignment *P*-value is computed based on the *Q*-score of a particular alignment and the total distribution of *Q*-scores in the population of pocket pairs. In detail, we fit the distribution of all the *Q*-scores by an extreme value distribution (EVD) and estimate the parameters of the distribution function as,, and. Then, the *P*-value for an alignment with *Q*-score is given by

$$ \begin{array}{l}P\left(s>x\right)=1-{e}^{-{\left[1+k\left(\frac{x-\mu }{\sigma}\right)\right]}^{-1/k}}\\ {}\end{array} $$

In this work, the significance threshold of the structure similarity *P*-value is set to 0.05.

### Classifying RNA-binding pockets via similarity-network-based clustering

We embed the overall similarities among pockets into a pocket similarity network built by linking an edge between any two pockets with significant structure similarity. The RNA-binding pockets are then clustered into groups based on the topological structure of the constructed similarity network. By employing a fast community detection algorithm [45], we decompose the network into several communities, each of which represents a cluster of structurally similar RNA-binding pockets. Essentially, the community detection method in the network is a hierarchical clustering with the advantages of improving the distinguishing measures between groups via the sparseness of the pocket similarity matrix [45, 46]. After the network decomposition, we detect the major groups in these clusters as the local structure classification of RNA-binding pockets. The structure patterns in each pocket group are then identified and investigated.

### Extracting consistent multiple structure alignments

Underlying each pocket group, the pairwise alignments of any two pockets might be inconsistent with each other. To obtain a consistent multiple structure alignment for all pockets in a group, we develop a novel multiple structure alignment algorithm based on the pairwise alignments of SAMO. Supposing that residue of pocket is aligned with the residue of pocket in the output of SAMO, we denote ; otherwise. The aim of multiple pocket alignment is to derive a consensus structure from these pairwise alignments. The problem can be formulated as an optimization problem as follows:

$$ \begin{array}{l} \max \kern0.28em {\displaystyle \sum_{1\le i,j\le M}{\displaystyle \sum_{1\le k,l,c\le N}{\delta}_{ijkl}{x}_{ikc}{x}_{jlc}}}\\ {}s.t.\kern2em {\displaystyle \sum_{1\le c\le N}{x}_{ikc}}\kern0.5em \le 1,\kern1.5em 1\le i\le \kern0.5em M,1\le \kern0.5em k\kern0.5em \le N\\ {}\kern3.5em {\displaystyle \sum_{1\le k\le N}{x}_{ikc}}\kern0.5em \le 1,\kern1.5em 1\le i\le \kern0.5em M,1\le \kern0.5em c\kern0.5em \le N,\end{array} $$

where *x*_*ikc*_ denotes whether residue *k* in pocket *i* is matched to residue *c* of the consensus structure. *M* and *N* refer to the number of pockets in a group and the residue length of the pocket, respectively. The objective function is to maximize the number of aligned residue pairs consistent with the consensus structure. The constraints guarantee one-to-one residue matching between any pocket and the consensus structure. The optimization problem is solved using a greedy strategy. We first identify a seed of three pockets in a group with maximal consistent aligned residue pairs. Then, the other pockets are greedily added to the seed to maximize the objective function by solving a maximum weight matching problem [47]. The seed is iteratively revised according to the optimal matching and regarded as the output consensus alignment of pockets after the final iteration.

### Coordinating RNA-binding pockets with domains and proteins

To detect the upper-level structure characteristics of RBPs that contain these grouped pockets, we coordinate and map the RNA-binding pockets with their parent RBPs. The sequence domains and global structures where the pockets are located are identified accordingly. A “bottom-up” hierarchical model of RNA-binding structures, from pockets and domains to global tertiary structures, is built up. Joint analyses of their structural complementarity in protein-RNA recognition are then performed. Here, the networks are illustrated using Cytoscape (http://www.cytoscape.org), and the structures are depicted using Pymol (http://www.pymol.org).

## Results and discussion

### RNA-binding pocket groups

**Fig. 2:**
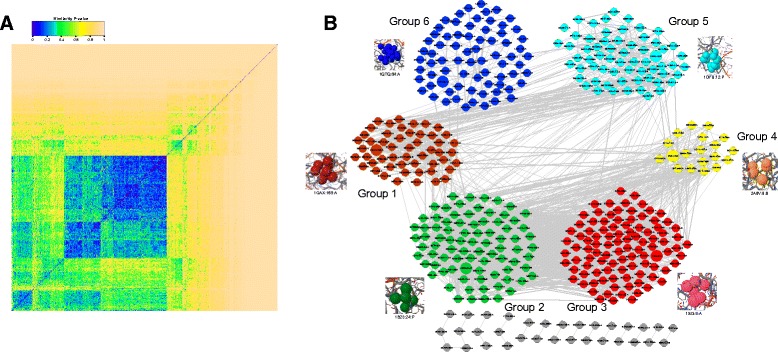
The global view of pocket similarity and the identified pocket groups in the RNA-binding pocket similarity network. **a**The heatmap of the structure similarities of RNA-binding pockets on the protein surface. **b** The pocket similarity network with different colored and numbered groups. A 3D pocket structure is displayed for a representative pocket of each of six major groups

Based on the pocket similarity network modeling the structural similarities among RNA-binding pockets, a community detection algorithm is employed to decompose the network into smaller groups of similar pockets. Figure 2 illustrates the heatmap of global similarities among these RNA-binding pockets and the major identified pocket groups via a network-based clustering strategy.

In Fig. 2(a), the significantly similar pockets form several blue blocks. We find that the near half of RNA-binding pockets cannot identify their significantly similar partners (yellow parts). This result indicates the diversity and flexibility of RNA-binding local structures on protein surfaces. The feasible space of RNA-binding local structures is very large, and many different shapes can perform RNA-binding functions on protein surfaces. More interestingly, a central block can be found in the global heatmap shown in Fig. 2(a). The results provide evidence of the consistency in RNA-binding local structures; that is, many pockets can find matching partners with similar shapes. The diversity and consistency of the clustering results reveal the complexity of the mechanism and structural space of local protein regions for recognizing RNA.

We focus on the significantly similar pocket pairs with *P*-value < 0.05 and build up the pocket similarity network by linking similar pockets. Figure 2(b) illustrates the pocket similarity network and the underlying communities. We identify the topological communities that naturally cluster the pockets into similar groups. Six major pocket groups are identified from the pocket similarity network. Accordingly, the RNA-binding pockets are classified into the six pocket groups numbered in Fig. 2(b). The pockets in a group are illustrated in the same color. For simplicity, the isolated pockets that lack any significantly similar partner are not displayed, and the few pockets not belonging to the six groups are colored as gray nodes. In each group, a representative pocket of the highest degree is presented as a larger node in the network, with a 3D pocket structure nearby. The local 3D structures demonstrate the structural patterns of pocket residues, which are formulated into binding pockets for interacting with RNA molecules. In our non-redundant RNA-binding proteins, the six pocket groups establish the main structure templates of RNA recognition occurring on protein surfaces. Thereafter, we converge our further analyses on the six identified RNA-binding pocket groups.

For instance, 79 pockets are clustered in Group 1, and pocket 1GAX:168:A (protein_ID:pocket_ID:chain_ID) is the representative pocket of this group. The middle number refers to the pocket ID in the protein chain. Several pockets in the RBP, e.g., 1P6V:A, are classified into the same group, e.g., 1P6V:38:A, 1P6V:43:A, 1P6V:49:A and 1P6V:55:A, while some pockets in the same protein are classified into other groups, e.g., 1P6V:33:A in Group 2. This finding indicates that the pockets in the same protein are not always classified into the same group.

### Patterns of RNA-binding pocket groups

**Fig. 3:**
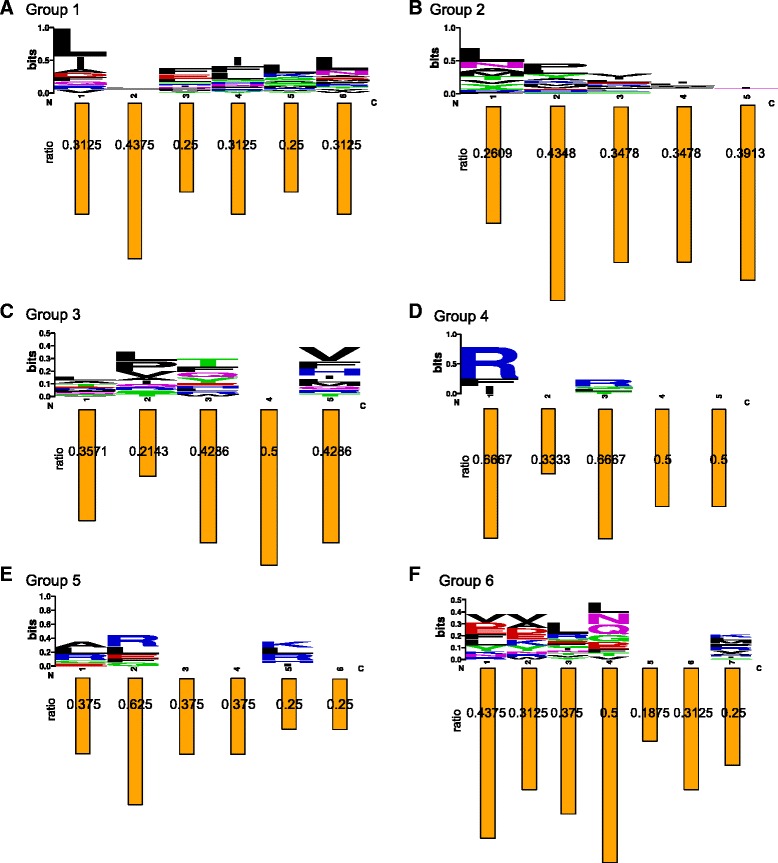
The sequence motif logos of six pocket groups and the ratios of active RNA-binding residues in each corresponding position. The sequence motif logos and bar graphs are drawn based on the results of the multiple structure alignment shown in Additional file 1: Figure S1

The sequence patterns of RNA-binding pocket groups are investigated by concatenating the component amino acids for each pocket, as illustrated in Additional file 1: Figure S1 and Fig. 3. The residues of a pocket are scattered and unordered in the parent protein sequence. That is, they are neighbors on the protein surface from a spatial perspective but not in the primary sequence, and they are not in the same sequential order in different proteins since the structure alignments are obtained by the non-sequential-order structure alignment method.

For each group, we extract a multiple structure alignment, and the sequences of some representative pockets are exhibited in Additional file 1: Figure S1. The original residue positions in the parent protein sequences are shown on the right top of each residue individually. The sequence motifs after multiple structural alignments are illustrated by the sequence logos in Fig. 3. The ratios of active RNA-binding residues in each position are also shown in positions corresponding to the locations. The patterns shown in the sequence motifs are not very significant, and the active binding residues are not obviously enriched in specific positions. This result implies that the functions of binding pockets are not simply determined by the primary protein sequence or single residues. Instead, the binding pockets should be studied as an undivided whole local structure, from a systematic perspective.

Note that these proteins are selected without sequence similarity, and the pocket similarities are measured by the non-sequential-order structure alignment algorithm. There is also no requirement of global structure similarity. Although there is no significant similarity among the protein sequences, global structures, and even the concatenated pocket sequences, the local structure similarity among pockets determines the functionality of RNA-binding. There is no significant structure similarity across the six pocket groups. Different groups contain their own spatial patterns. The pocket structures, such as the representative structures shown in Fig. 2(b), can be regarded as the structure motifs of binding RNAs. The structure patterns indicate that the pockets provide the detailed structural environments and complementary components for recognizing their RNA partners.

The low sequence similarity underlying each group also indicates few enriched residues playing crucial roles in RNA-binding. Instead of sequence equivalence, a few residues adopt a local spatial shape that interacts with RNA on the protein surfaces. Several specific amino acid residues play essential roles in one pocket, and several pockets cooperate with each other in the binding. For example, the RBP 1GAX:A contains four RNA-binding pockets, of which 1GAX:115:A, 1GAX:118:A and 1GAX:168:A are clustered in Group 1 and 1GAX:236:A in Group 2. The pocket 1GAX:168:A is the hub pocket shown as the representative 3D structure in Fig. 2(b). From the sequences of Group 1, we can easily find that leucine is enriched in these pockets. The five residues in each pocket construct a cavity on the protein surface and are not ordered in the primary protein sequence. While they form a certain structural complementarity for recognizing specific RNA, their local spatial patterns cause the interaction to occur at the atomic and molecular levels.

### Classification of RNA-binding domains and global structures

In the former sections, we constructed the pocket similarity network by comparing the local RNA-binding cavities in an all-against-all manner. Using network-based clustering, we identified six major groups of RNA-binding pockets, which are local spatial patterns and structural templates involved in the recognition of RNA. Obviously, these local structure motifs occur in their parent domains and proteins. From a hierarchical perspective, from the pocket and domain to the global structure, it is valuable to reveal the high-level upstream similarity among these parent RNA-binding domains and global structures.

Checking the sequence components of these RBPs, we find that most of the RNA-binding pockets are located in certain RNA-binding domains. Most of the RBPs (102/158) contain at least one annotated RNA-binding domain, some of which are shown in Additional file 1: Table S1. The Pfam [17] domain and superfamily annotations are illustrated, having been generated using a hidden Markov model. As shown in Supplementary Table S1, most of the proteins can be classified in the Pfam domain superfamily clans, while several domains such as ‘Elongation factor Tu C-terminal domain’, ‘Elongation factor SelB, winged helix’ and ‘PAZ domain’ still cannot currently find their corresponding clans (shown at the bottom of Supplementary Table S1). Some proteins also contain several domains. For instance, protein 1U0B:B has ‘tRNA synthetases class I (C) catalytic domain’ and ‘DALR domain’. Both are related to tRNA synthesis. The consistency and diversity of the domains in RBPs indicate the functional complexity of protein-RNA binding events, such as ‘tRNA synthetases’, ‘RNA polymerase’, ‘RNA recognition motif’, ‘Ribonuclease’, and ‘Zinc finger’.

**Fig. 4:**
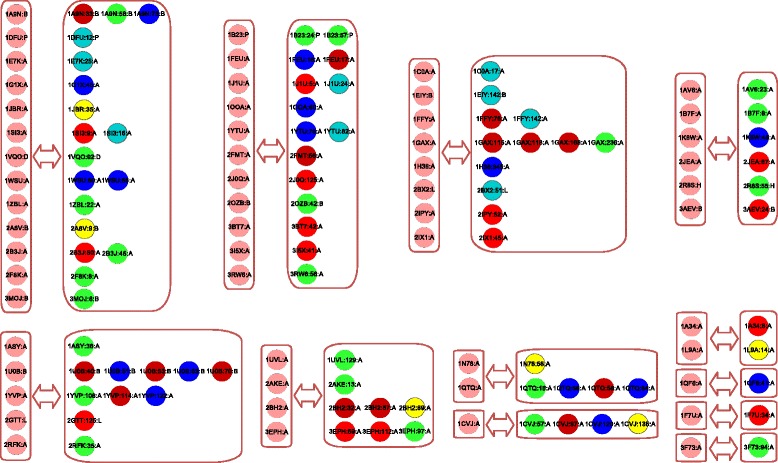
The corresponding classifications between protein groups and pocket groups. The RBP groups in the frames were clustered based on a protein similarity network, similarly to the pocket groups. When a pocket is contained in a protein, it is placed on the same row to the right of the corresponding protein. The pockets in the same pocket group are shown in the same color.

Although the RBPs lack significant sequence similarity with each other (less than 25%), some of them contain the same RNA-binding domains. For instance, both proteins 1YTU:A and 3 F73:A contain the ‘Piwi domain’. The same domain underlying two proteins determines their structure similarity. As shown in Fig. 4, they are clustered in the same protein group according to their global structure similarity. This finding implies that the domain units are also important for performing RNA-binding functions in the global protein structures. Some proteins such as 1F7U:A contain several domains, which indicates that they will perform multiple functional roles with RNAs. Different parts of the protein structure perform different RNA-binding-related activities. It is already known that the RNA-binding domains specify the sequence profiles and patterns of the local structure basis of protein-RNA recognition [1, 12].

The pockets are local regions and particular subparts of the RNA-binding domains. Based on the similarity network framework, we identified the major groups of RNA-binding local structures, which illustrates that the RNA-binding domains fold into certain local 3D structure patterns, and the resulting pockets formed on protein surfaces facilitate the local sites and environments for binding to RNA partners. We should analyze the RNA-binding local structures on protein surfaces to decipher their functional importance and complexity, as well as to identify specific RNA-binding structure motifs [24].

Note that sequence alignment cannot reveal homologous relationships among these RNA-binding proteins. The classification of global protein structures provides a higher-level overview of the relationships among proteins in the recognition of RNA. In a network-based framework similar to pocket group identification, we build a protein similarity network for describing the global structure similarities among these RBPs. That is, one node represents one protein, and when the global structure similarity between two proteins exceeds a given threshold of significance (*P*-value < 0.05), two nodes are linked by an edge. In this way, we implement a rapid community structure detection method to classify the RBPs into 12 groups. Figure 4 illustrates these identified protein groups with some representative proteins and their child RNA-binding pockets. From the correspondence between two levels of elements, we find no strict consistency between protein groups and pocket groups. In Fig. 4, the pockets are uniformly shown in their respective group colors. The pockets of the same-group proteins are not always classified into the same pocket group, which demonstrates the complexity of RNA-binding functionality from the perspective of global and local structure components. In fact, the pockets of the same-group proteins are flexibly categorized into diverse pocket groups with different colors, and vice versa.

Although similar RBP structures contain similar RNA-binding pockets, we find that there exist inconsistent correspondences between proteins and pockets. For instance, proteins 1ASY:A and 2GTT:L are in the same protein group, but their RNA-binding pockets are not in the same group. In another example, protein 1QTQ:A has four pockets, 1QTQ:16:A, 1QTQ:56:A, 1QTQ:58:A and 1QTQ:64:A, that are located in three different pocket groups. These examples imply that RNA-binding events occur mainly in the specific local structure environments of pockets. The protein-RNA recognition is a very specifically functional event in these local structures. Thus, the results of this study confirm that RNA-binding function in proteins is more conserved in the local structure space than in the global structure space of RBPs.

Thus far, there are still no direct functional annotations of these local structure pockets on protein surfaces. To perform a functional enrichment analysis of these pocket groups, we use the gene ontology (GO) [48] molecular functions of their parent proteins to implement functional annotation analysis via DAVID [49]. For illustration, Table 1 lists the top 5 significant GO molecular functions in the six pocket groups. The full lists with significance *P*-value corrections are available on our website. According to Table 1, we find that some RNA-binding-related functions are enriched in these pocket groups, such as ‘nucleotide binding’ and ‘forming aminoacyl-tRNA and related compounds’. The underlying functions in each pocket group indicate the functional importance of these analyzed pockets in binding RNAs. An important research topic in the near future is to determine the concrete functions of these binding pockets. Functional annotations of the protein surfaces will greatly help to decipher the code-like principles of protein-RNA interaction and recognition [50].

**Table 1:** Functional analysis of the GO molecular functions of the six pocket groups. ‘Group’ is the pocket group ID. ‘Term’ refers to the GO molecular functions with their descriptions. Some pockets in the group are listed as ‘Representative pockets’. ‘*P*-value’ is the enrichment significance. Note that the functions of the parent proteins are used to implement the calculations because there are no GO annotations of these pockets thus far. For each term, ‘Count’ refers to the number of proteins containing the GO term in the ‘Population total’ background RBPs. ‘List total’ refers to the number of proteins in one group. ‘Population hits’ is the number of proteins annotated with the term. (In DAVID, there are slightly different numbers in ‘List total’ and ‘Population total’ for one group when calculating a specific GO term). For conciseness, we list only the top 5 terms in each group; the complete tables are available from our website

Group	Term	Count	List total	Population hits	Population total	*P*-value	Representative pockets
Group 1	GO:0046914 ~ transition metal ion binding	10	32	17	123	0.032977	1U0B:40:B, 1UVL:183:A, 2BH2:32:A, 2IPY:137:A, 3EPH:130:A
	GO:0043167 ~ ion binding	11	30	27	119	0.071152	1U0B:40:B, 1UVL:183:A, 2BH2:32:A, 3EPH:130:A
	GO:0046872 ~ metal ion binding	11	30	27	119	0.071152	1U0B:40:B, 1UVL:183:A, 2BH2:32:A, 3EPH:130:A
	GO:0043169 ~ cation binding	11	30	27	119	0.071152	1U0B:40:B, 1UVL:183:A, 2BH2:32:A, 3EPH:130:A
	GO:0016779 ~ nucleotidyltransferase activity	5	30	24	123	0.097784	1H38:166:A, 1UVL:183:A
Group 2	GO:0000166 ~ nucleotide binding	27	52	44	119	0.005401	1A9N:58:B, 1ASY:38:A, 1B23:24:P, 1C0A:13:A, 1CVJ:57:A, 1QTQ:16:A, 1UVL:129:A, 2AKE:13:A, 2F8K:8:A, 3EPH:97:A, 3MOJ:8:B, 3RW6:56:A
	GO:0032559 ~ adenyl ribonucleotide binding	20	52	30	119	0.008157	1ASY:38:A, 1C0A:13:A, 1QTQ:16:A, 2AKE:13:A, 3EPH:97:A, 3MOJ:8:B
	GO:0005524 ~ ATP binding	20	52	30	119	0.008157	1ASY:38:A, 1C0A:13:A, 1QTQ:16:A, 2AKE:13:A, 3EPH:97:A, 3MOJ:8:B
	GO:0004812 ~ aminoacyl-tRNA ligase activity	11	52	15	119	0.043756	1ASY:38:A, 1C0A:13:A, 1QTQ:16:A, 2AKE:13:A
	GO:0046914 ~ transition metal ion binding	14	52	21	119	0.045022	1UVL:129:A, 1ZBL:22:A, 2B3J:46:A, 3EPH:97:A
Group 3	GO:0030554 ~ adenyl nucleotide binding	18	51	30	119	0.04838	1F7U:34:A, 1QTQ:25:A, 3EPH:112:A
	GO:0001882 ~ nucleoside binding	18	51	30	119	0.04838	1F7U:34:A, 1QTQ:25:A,2A8V:16:B3EPH:112:A
	GO:0001883 ~ purine nucleoside binding	18	51	30	119	0.04838	1F7U:34:A, 1QTQ:25:A,2A8V:16:B3EPH:112:A
	GO:0032553 ~ ribonucleotide binding	20	51	35	119	0.061405	1F7U:34:A, 2A8V:16:B, 3EPH:112:A
	GO:0032555 ~ purine ribonucleotide binding	20	51	35	119	0.061405	1F7U:34:A, 2A8V:16:B, 3EPH:112:A
Group 4	GO:0046914 ~ transition metal ion binding	6	14	21	119	0.052374	1U0B:78:B, 1UVL:73:A, 2BH2:89:A, 3EPH:50:A
	GO:0043167 ~ ion binding	6	14	27	119	0.13911	1UVL:73:A, 2BH2:89:A
	GO:0043169 ~ cation binding	6	14	27	119	0.13911	1UVL:73:A, 2BH2:89:A
	GO:0046872 ~ metal ion binding	6	14	27	119	0.13911	1UVL:73:A, 2BH2:89:A
	GO:0000166 ~ nucleotide binding	8	14	44	119	0.151441	1CVJ:135:A, 1 N78:58:A, 2A8V:9:B
Group 5	GO:0016876 ~ ligase activity, forming aminoacyl-tRNA and related compounds	9	42	15	119	0.08969	1C0A:17:A, 1EIY:142:B, 1J1U:24:A,2AKE:32:A
	GO:0004812 ~ aminoacyl-tRNA ligase activity	9	42	15	119	0.08969	1C0A:17:A, 1EIY:142:B, 1J1U:24:A, 2AKE:32:A
	GO:0046872 ~ metal ion binding	12	42	27	119	0.287759	1EIY:142:B, 1YTU:82:A
	GO:0032553 ~ ribonucleotide binding	14	42	35	119	0.422562	1C0A:17:A, 1EIY:142:B, 1J1U:24:A
	GO:0030554 ~ adenyl nucleotide binding	12	42	30	119	0.46644	1C0A:17:A, 1EIY:142:B, 1J1U:24:A
Group 6	GO:0046914 ~ transition metal ion binding	10	41	21	119	0.229275	1U0B:49:B, 1UVL:243:A, 2BH2:56:A, 2IPY:165:A, 3EPH:116:A
	GO:0000166 ~ nucleotide binding	18	41	44	119	0.245078	1A9N:73:B, 1CVJ:120:A
	GO:0008173 ~ RNA methyltransferase activity	4	41	6	119	0.32238	2BH2:56:A, 3BT7:93:A
	GO:0046872 ~ metal ion binding	11	41	27	119	0.416938	1YTU:76:A
	GO:0043167 ~ ion binding	11	41	27	119	0.416938	1YTU:76:A

### Case study of RNA-binding structure motifs

**Fig. 5:**
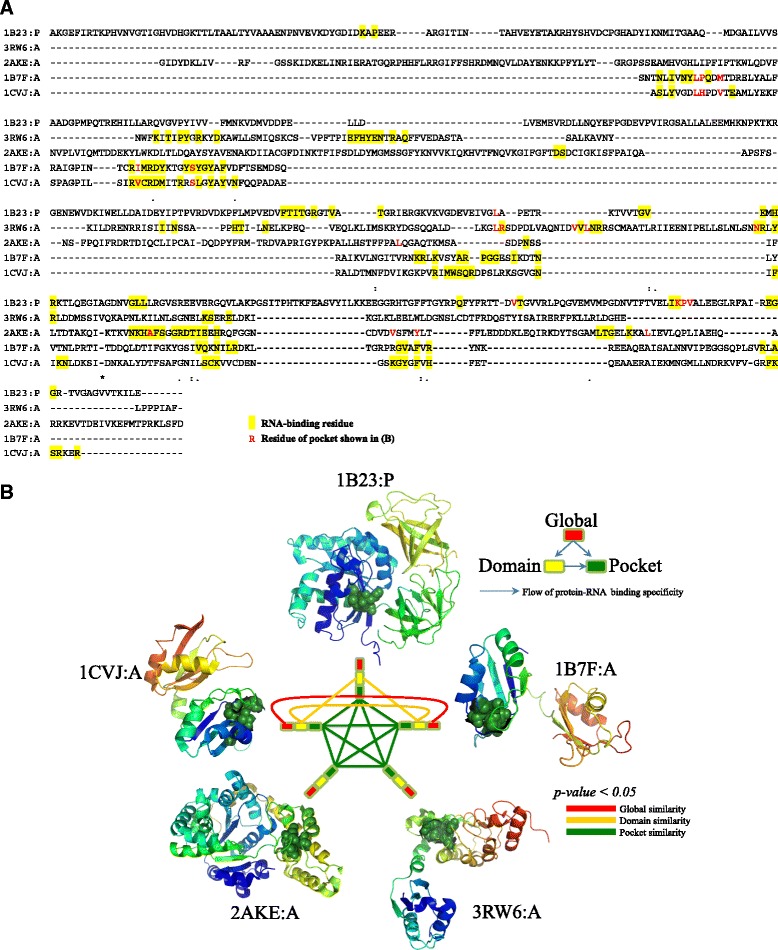
Details of an example local structure motif in RBPs. **a** The multiple sequence alignment of five RBPs with the locations of RNA-binding pockets and residues. **b** The similarities among global protein structures, sequence-based domains and local pockets. Significant similarity is represented by an edge in different colors corresponding to different levels, i.e., green for pocket, yellow for domain and red for global structure. Some proteins contain similar domains, while others do not. All five proteins contain similar RNA-binding pockets serving as local structure motifs, which determine the similar RNA-binding functionality

We classified the RNA-binding pockets by a similarity-network-based framework via all-against-all structure comparisons. The correspondence between different levels of RNA-binding components such as sequences, structures, domains, pockets, and residues has been analyzed accordingly. For a detailed illustration of the identified RNA-binding structure motifs, Fig. 5 shows a case study of structure motifs in five RBPs, i.e., 1B23:P, 1B7F:A, 1CVJ:A, 2AKE:A, and 3RW6:A. Figure 5(a) is the multiple sequence alignment, and Fig. 5(b) illustrates the comparison of the pockets, domains, and global structures.

In Fig. 5(a), there is no significant sequence similarity in the sequence alignments. The RNA-binding amino acids in contact with RNA nucleotides are shown in yellow. The RNA-binding pockets are shown by red residues, which are scattered irregularly in the primary sequences. There is only one pair of proteins with significant global structure similarity, i.e., 1B7F:A and 1CVJ:A. They are connected by a red edge in the center of Fig. 5(b). Three pairs of proteins, i.e., 1B23:P-1CVJ:A, 1B7F:A-1CVJ:A, and 1B23:P-1B7F:A, contain similar domains, which are linked by yellow edges. Among them, the pair 1B7F:A-1CVJ:A contains the same ‘RNA recognition motif’ (RRM) domain. The RRM domain is the main substructure of their global 3D structures. However, the RNA-binding domains in the five proteins are not all similar to each other. Although proteins 1B23:P, 2AKE:A and 3RW6:A contain no such RRM domain, they contain the domains ‘Elongation factor Tu GTP-binding domain’, ‘tRNA synthetases class I’ and ‘Leucine Rich Repeat′, respectively. The domain similarities described by the yellow edges of 1B23:P-1B7F:A and 1B23:P-1CVJ:A 1B23:P and 1B7F:A are determined by the structure similarities between domain “Elongation factor Tu domain” (in 1B23:P) and RRM domain (in 1B7F:A and 1CVJ:A). The domain has been shown to be critically involved in RNA-binding [51].

Notably, the RNA-binding pockets in these proteins are significantly similar, shown as a green clique in the center of Fig. 5(b). It is easy to understand that similar global protein structures contain similar pockets, such as 1B7F:A and 1CVJ:A. Analogously, similar domains imply similar pockets, such as 1B23:P and 1B7F:A. These similarities of global and domain structures imply similar RNA-binding functionality. The RNA-binding pockets are the determinants of their specificity. When the similarity both between global protein structures and both between domains disappears, these proteins still can contain similar pockets. This finding directly proves that different global structures and different domains can contain similar local structure binding motifs, i.e., the RNA-binding pockets on protein surfaces. We align these pockets purely by their 3D structures, and they have no direct relationships with sequence domains and secondary structures due to their discontinuous residue locations in the primary protein sequence. They are flexibly located on protein surfaces that can cover different domains and across various secondary structure elements. The annotations show that these five proteins contain the function of ‘GO:0000166 ~ nucleotide binding’ [48]. Fig. 5 confirms that regardless of whether proteins contain similar global structures or domains, if they have similar pockets that can recognize RNA molecules and bind to them, the proteins will participate in the RNA-binding functionality. The pocket performs the specific RNA-binding functions and works as a functional unit interacting with RNA. The results also clearly suggest that the identification of RNA-binding structure motifs should involve extracting the structure patterns in these regional surfaces, instead of from global protein structures and domains.

Figure 5(b) also demonstrates a case of an RNA-binding structure motif. The functional flow is from ‘global’ protein to ‘domain’ to ‘pocket’ as shown in the top-right legend of Fig. 5(b). The similarities of global structures and domains are not regularly necessary requirements for binding RNA. The structure motifs, in the form of pockets on protein surfaces, endow the protein with the ability of RNA-binding. This finding indicates that the flexibility of the global structure and the strict requirements for the local structures outline valuable strategies for detecting the possibility of certain targets in drug design techniques. The importance of local structure motifs is strengthened by our proposed similarity-network-based framework of structure comparison and classification.

## Conclusions

In this paper, we conducted a systematic analysis of RNA-binding pockets on protein surfaces to reveal the binding structure motifs in protein-RNA recognition. After extracting the large-scale RNA-binding pockets on protein surfaces, we improved our non-sequential-order structure alignment algorithm, SAMO, to better measure local similarities and proposed a similarity-network-based framework to cluster the pockets into similar groups. Then, in these groups, we developed a multiple structure alignment strategy to identify their consensus alignment and coordinate the correspondences between the RNA-binding pockets and their parent proteins. The multi-level analyses demonstrate the RNA-binding structure neighbors in different spaces of protein components, i.e., global structure, domain and pocket. Using the similarity-network-based strategy, we revealed the major groups of RNA-binding structure motifs in the form of pockets and their patterns of RNA-binding.

Specifically, we identified the protein-RNA binding pockets in non-redundant RBPs. By implementing a similarity-network-based clustering method, we identified the major classes of RNA-binding pockets and the corresponding classes of the parent sequence domains and global structures. The similarities in these RNA-binding domains and global proteins are not inherently consistent with the similarities among RNA-binding pockets. Very different proteins and domains might contain similar RNA-binding pockets. These findings provide direct evidence for the importance of binding pockets on protein surfaces in the protein-RNA recognition. The results highlight that the RNA-binding pockets are the functional units providing structural specificity for recognizing RNAs. The classified binding pockets and their structure patterns are potentially valuable in RNA-interaction-related protein design and engineering. As increasing numbers of structures of protein-RNA complexes become available, the set of identified pocket groups will be expanded, and some novel patterns will be revealed using the classification strategy. The proposed framework of identifying RNA-binding structure motifs can be flexibly generalized and extended to study other local structures, such as ligand-binding residues and protein-interacting hotspots.

## Additional file

Additional file 1: Table S1. Some RBPs and their corresponding domains and families. **Table S2.** The overlapping number of pockets in the six groups. **Table S3.** Functional GO annotations for the five proteins in the case study. **Figure S1.** The dendrogram of pocket groups and the sequences of the RNA-binding pockets. (PDF 314 kb)

## Abbreviations

3D: Three dimensional
FDR: False discovery rate
GO: Gene ontology
ID: Identifier
PDB: Protein data bank
RBP: RNA-binding proteins
RMSD: Root mean square deviation
RNA: Ribonucleic acid
RRM: RNA recognition motif
SAMO: Structural alignment by multi-objective optimization
