# Supplementary material for: Structure alignment-based classification of RNA-binding pockets reveals regional RNA recognition motifs on protein surfaces
Source: BMC Bioinformatics. 2017 Jan 11;18:27. doi: 10.1186/s12859-016-1410-1 (PMC5225598; doi:10.1186/s12859-016-1410-1)
Supplement: Additional file 1: Table S1. — Some RBPs and their corresponding domains and families. Table S2. The overlapping number of pockets in the six groups. Table S3. Functional GO annotations for the five proteins in the case study. Figure S1. The dendrogram of pocket groups and the sequences of the RNA-binding pockets. (PDF 314 kb) [file 12859_2016_1410_MOESM1_ESM.pdf]

Supplementary Materials

Structure alignment-based classification of RNA-binding pockets reveals regional RNA recognition motifs on protein surfaces

Zhi-Ping Liu[1], Shutang Liu[1], Ruitang Chen[2], Xiaopeng Huang[3,4,5], Ling-Yun Wu[3,4,5,*]

[1]*Department of Biomedical Engineering, School of Control Science and Engineering, Shandong University, Jinan, Shandong 250061, China*

[2]*Department of Computer Science, Stanford University, Stanford, CA 94305, USA*

[3]*Institute of Applied Mathematics, Academy of Mathematics and Systems Science, Chinese Academy of Sciences, Beijing 100190, China*

[4]*National Center for Mathematics and Interdisciplinary Sciences, Chinese Academy of Sciences, Beijing 100190, China*

[5]*University of Chinese Academy of Sciences, Beijing 100049, China*

**Correspondence: lywu@amss.ac.cn*

Table S1: Some RBPs and their corresponding domains and families. For conciseness, only partial lists are shown for the complicated correspondences between domains and proteins. 'Clan ID' refers to the domain's super family with description. 'Protein' indicates the RBP(s) containing the domain (with 'Domain ID' and its 'Description'). The full lists are available at our website.

Clan ID	Description	Domain ID	Description	Protein
CL0007	K-Homology (KH) domain superfamily	PF00013	KH domain	3AEV:B
CL0027	RNA-dependent RNA polymerase	PF30561	RNA-dependent RNA polymerase	1UVL:A
CL0039	HUP - HIGH-signature proteins, UspA, and PP-ATPase	PF38198	tRNA synthetases class I (I, L, M and V)	1FFY:A;1GAX:A
		PF00579	tRNA synthetases class I (W and Y)	1J1U:A;2AKE:A
		PF00749	tRNA synthetases class I (E and Q), catalytic domain	1N78:A;1QTQ:A
		PF00750	tRNA synthetases class I (R)	1F7U:A
		PF01406	tRNA synthetases class I (C) catalytic domain	1U0B:B
CL0040	Class II aminoacyl-tRNA and Biotin synthetases	PF00152	tRNA synthetases class II (D, K and N)	1ASY:A;1C0A:A
		PF00587	tRNA synthetase class II core domain (G, H, P, S and T)	1QF6:A
		PF01358	Poly A polymerase regulatory subunit	1AV6:A
		PF01409	tRNA synthetases class II core domain (F)	1EIY:B
		PF05958	tRNA (Uracil-5-)-methyltransferase	2BH2:A;3BT7:A
CL0178	PUA/ASCH superfamily	PF01472	PUA domain	2RFK:A
		PF09157	Pseudouridine synthase II TruB, C-terminal	1K8W:A
CL0219	Ribonuclease H-like superfamily	PF00075	RNase H	1ZBL:A
		PF02171	Piwi domain	1YTU:A;3F73:A

CL0221	RRM-like clan	PF00076	RNA recognition motif (a.k.a. RRM, RBD, or RNP domain)	1B7F:A;1CVJ:A
		PF13893	RNA recognition motif (a.k.a. RRM, RBD, or RNP domain)	1A9N:B
CL0258	DALR superfamily	PF05746	DALR anticodon binding domain	1F7U:A
		PF09190	DALR domain	1U0B:B
CL0298	tRNA-binding arm superfamily	PF10458	Valyl tRNA synthetase tRNA binding arm	1GAX:A
CL0329	Ribosomal protein S5 domain 2-like superfamily	PF01138	3' exoribonuclease family, domain 1	2JEA:A
CL0458	Class II aaRS Anticodon-binding domain-like	PF03129	Anticodon binding domain	1QF6:A
-	-	PF03143	Elongation factor Tu C-terminal domain	1B23:P
-	-	PF09107	Elongation factor SelB, winged helix	1WSU:A
-	-	PF02170	PAZ domain	1SI3:A

Table S2: The overlapping number of pockets in the six groups identified by the community-based clustering method used in this paper and the K-means method. The major pockets identified in one group by one method are predominantly allocated into two or three clusters identified by the other method.

Overlap	K-means Group 1	K-means Group 2	K-means Group 3	K-means Group 4	K-means Group 5	K-means Group 6
Group 1	0	0	3	15	2	32
Group 2	55	23	2	1	2	0
Group 3	15	84	1	0	0	0
Group 4	3	13	0	1	0	0
Group 5	0	0	3	31	3	26
Group 6	0	0	30	0	49	0

Table S3: Functional GO annotations for the five proteins in the case study.

Protein	Gene Ontology	Type
2AKE:A	GO:0000166~nucleotide binding	Molecular Function
	GO:0004812~aminoacyl-tRNA ligase activity	Molecular Function
	GO:0004830~tryptophan-tRNA ligase activity	Molecular Function
	GO:0005524~ATP binding	Molecular Function
	GO:0005737~cytoplasm	Cellular Component
	GO:0006418~tRNA aminoacylation for protein translation	Biological Process
	GO:0006436~tryptophanyl-tRNA aminoacylation	Biological Process
1B7F:A	GO:0003676~nucleic acid binding	Molecular Function
	GO:0000166~nucleotide binding	Molecular Function
	GO:0003723~RNA binding	Molecular Function
1CVJ:A	GO:0000166~nucleotide binding	Molecular Function
	GO:0003676~nucleic acid binding	Molecular Function
1B23:P	GO:0000166~nucleotide binding	Molecular Function
	GO:0003746~translation elongation factor activity	Molecular Function
	GO:0003924~GTPase activity	Molecular Function
	GO:0005525~GTP binding	Molecular Function
	GO:0005622~intracellular	Cellular Component
	GO:0005737~cytoplasm	Cellular Component
	GO:0006412~translation	Biological Process
	GO:0006414~translational elongation	Biological Process
3RW6:A	GO:0000166~nucleotide binding	Molecular Function
	GO:0003723~RNA binding	Molecular Function
	GO:0005634~nucleus	Cellular Component
	GO:0005737~cytoplasm	Cellular Component
	GO:0006406~mRNA export from nucleus	Biological Process

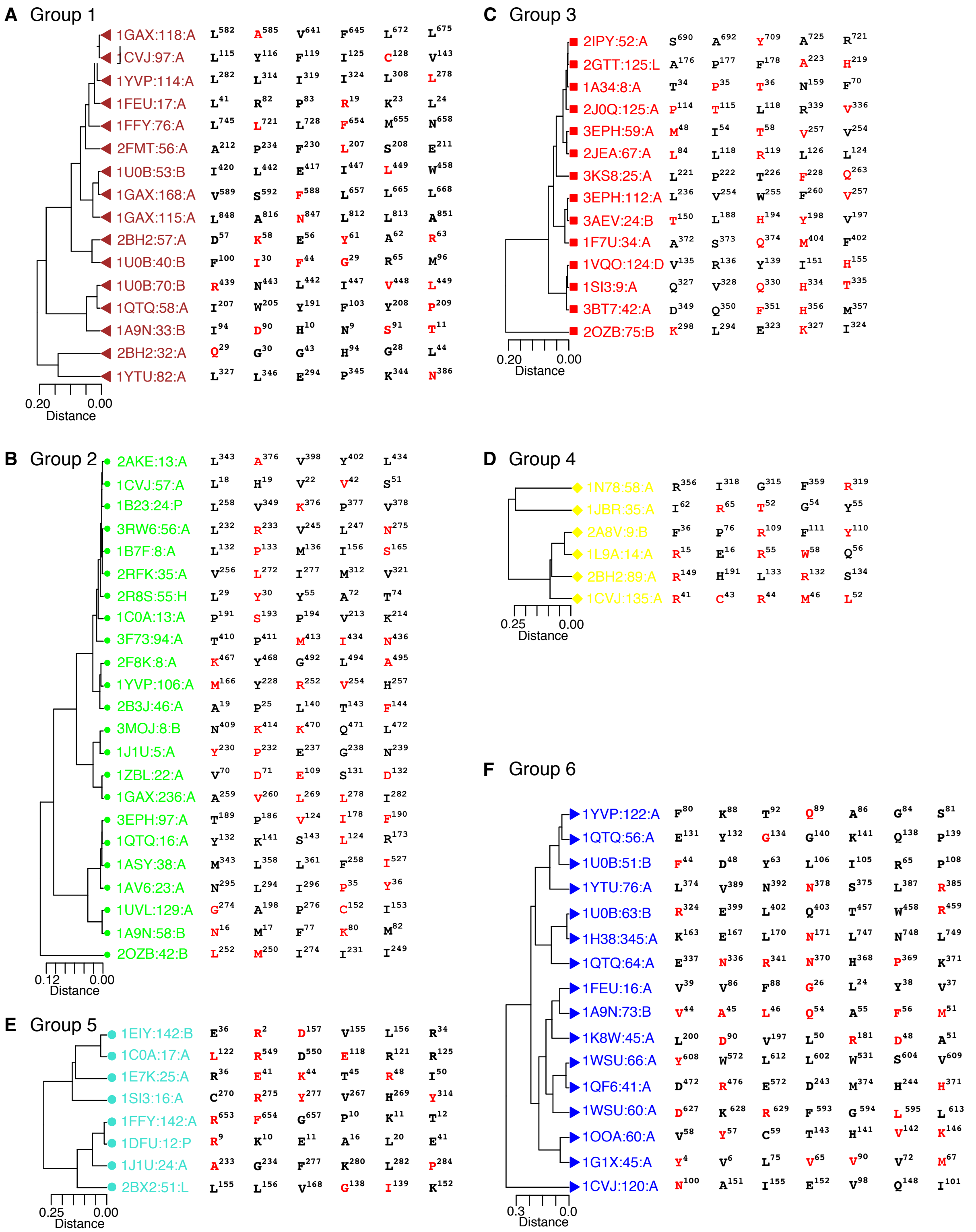

Figure S1: The dendrogram of pocket groups and the sequences of the RNA-binding pockets. The left of each subfigure shows the dendrogram of each of the six pocket groups. Different groups are displayed in different colors as in Figure 2 and 3. The concatenated residues of some representative pockets with the original sequential positions (right-top numbers) in the

protein sequences are shown on the right. The consensus sequences are optimally positioned via multiple structure alignments.
